# Characterizing the Profiles of Gram-Negative Bacterial Pathogens of Wound Infections and Their Drug-Resistance Disposition

**DOI:** 10.3390/microorganisms14051020

**Published:** 2026-04-30

**Authors:** Lorina Badger-Emeka

**Affiliations:** Microbiology Division, Department of Biomedical Sciences, College of Medicine, King Faisal University, Al-Ahsa 31982, Saudi Arabia; lbadgeremeka@kfu.edu.sa

**Keywords:** wound infections, Gram-negative, bacteria pathogens, antimicrobial resistance, antibiotics

## Abstract

Wound infections result from contamination of compromised skin following either intentional or accidental trauma. The failure of infected wounds to heal has a huge impact on global healthcare finances. For surveillance purposes, this investigation looks at wound infections and their susceptibility to antibiotics. Data obtained from the microbiology laboratory for the years 2014 and 2019 included wound characteristics, patient demographics, and causative bacteria pathogen. Also retrieved from an −80° C freezer were 270 Gram-negative bacteria isolates from wounds that formed part of patient care. Vitek Compact 2 was used for bacteria IDs and AST testing. Wound swabs were the majority (74.07%), followed by bedsore samples (12.22%). Others were tissue cultures (6.3%), skin swabs (3.7%), necrotizing fasciitis (1.48%), foot swabs (1.10%), and cervical wounds (1.11%). Isolated pathogens included *Pseudomonas aeruginosa* (33.6%), *Escherichia coli* (24.78%), *Acinetobacter baumannii* (21.85%), *Klebsiella pneumoniae* (17.65%), *Proteus mirabilis* (1.7%), and *Morganelli morganii* (0.41%). Most isolates had become MDR after 5 years, with extensive (100%) resistance to β-lactam and fluoroquinolone. Only tigecycline and amikacin maintained their antimicrobial activity for the period with some bacteria species. Suitable therapeutic options were few, irrespective of the year of isolation, particularly among the ESKAPE isolates. Overall results demonstrate that after a 5-year period, about 75% of the isolates of the bacteria pathogens had become resistant to most of the antibiotics used for their management.

## 1. Introduction

Wound infections arise from the contamination of skin injuries and the underlying tissues by microorganisms that include bacteria, viruses, or fungi. Common types of wounds can be categorized to include bedsores, foot wounds, infections of surgical sites, and burns, among a wide range of others [1]. However, the process of wound healing is impeded by any form of microbial infection, thereby prolonging inflammation, which subsequently leads to tissue destruction [2]. It is reported that about 8.2 million people who were Medicare beneficiaries had wounds with or without infections, with an estimated Medicare cost that ranged from USD 28.1 to USD 96.8 billion for chronic and acute wounds [3]. Highest among these were expenses incurred in the treatment of surgical wounds and diabetic ulcers [3]. At the time of this report, the Medicare cost for outpatient wound infections (USD 9.9–USD 35.8 billion) had a higher trend when compared with those that of inpatients (USD 5.0–USD 24.3 billion). Globally, on an annual basis, the pain and trauma of wounds are endured by individuals around the world, a condition that is further complicated by infections [1].

Generally, wound infections are of public health concern in healthcare settings globally, including Saudi Arabia, with a huge impact on available financial resources, affecting the overall efficacy of the healthcare system. The burden of wound infections is yet to be expansively appraised, but is considered to be high in Saudi Arabia [4]. This is in view of the high prevalence of challenging health conditions that include obesity, ischemic heart disease, and diabetes among the Saudi Arabia populace. Effective and timely management of such infections will not only reduce the financial burden for treatment but also the resultant traumatic effects [5].

There is also the issue of caring for wounds at home by the untrained due to the influence of traditional medical views that not only impairs the wound-healing process but also increases the risk of developing infections [6,7]. To establish the appropriate treatment of resultant infections, clinicians should diagnose an infected wound and confirm through microbiological assay [8]. However, because such diagnostic procedures are usually time-consuming, empirical antibiotic treatment is instituted without adequate and conclusive diagnosis, thereby creating a greater clinical and economic burden by contributing to more AMR [9].

However, wounds can fail to heal, and this can be attributed to polymicrobial infections, as well as other factors that include patient comorbid conditions, which contribute to the sustainability of healthcare services. Moreover, there is also the rise of drug-resistant bacteria contributing to the prompt and adequate management of bacterial infections globally, thus leading to the long hospitalization and suffering of patients [10]. Saudi Arabia is a region with high antimicrobial resistance attributed to practices of physicians in the use of antimicrobials [11]. Regular monitoring of the antimicrobial susceptibility of bacteria pathogens is recommended as this could be a guide for antimicrobial therapeutic treatment for wound infections. This investigation seeks to bridge the gap in knowledge in this region by assessing the profiles of Gram-negative bacteria pathogens associated with wound infections and their disposition to antibiotics, providing information that could help guide the treatment of these recalcitrant wound pathogens.

## 2. Materials and Methods

### 2.1. Study Area, Samples, and Ethical Considerations

The study was conducted in Al-Ahsa, which is located between Dammam and Riyadh, in the southeast of Saudi Arabia. The research utilizes the available data in the Microbiology Division of the Department of Biomedical Sciences of the College of Medicine, King Faisal University. Retrieved data included demography (age and gender), date of isolation, sample type, and the source (ward) of origin. Wound infections were taken as those saved as wound swabs, tissue cultures, bedsores, skin ulcers, and necrotizing fasciitis. Only data that equally had the isolated bacteria pathogen stored in the −80 °C microbank were used for the investigation. The research protocol was approved by the Research Ethics Committee of King Faisal University, approval number KFU-REC-2026-JAN– ETHICS53. Humans were not involved in the study and are therefore exempted from informed consent, subject to provisions of the National Committee of Saudi Arabia Bioethics (kacst.gov.sa, Section 11).

### 2.2. Bacterial Isolates Retrieval and Confirmation of IDs

Gram-negative bacteria isolates that were preserved in MicrobankTM were retrieved from an −80 °C freezer. Preservation and retrieval of the bacteria isolates were undertaken according to the guidelines of the manufacturers (https://www.pro-lab-direct.com/v/vspfiles/microbank/microbank-wwp-portfolio.pdf, accessed 8 December 2025). Isolates were retrieved by plating out on MacConkey agar, cultured aerobically for 24 h at 37 °C. The overnight bacteria colony growths were plated out and cultured under the same conditions to obtain pure colonies that were used for the confirmation of bacteria identities with Vitek Compact 2 (BioMérieux, Marcy L’Etoile, France), applying GN-ID cards according to the guidelines of the manufacturers (https://www.epa.gov/sites/default/files/2017-01/documents/qc-22-04.pdf, accessed 8 December 2025). Briefly, pure overnight colony bacteria growths for each of the isolates were suspended in sterile 3 mL of 0.45% saline solution. Using a DensichekTM turbidity meter (BioMérieux Inc. DensichekTM™, Marcy L’Etoile, France), bacteria suspensions for each of the isolates were prepared to attain a turbidity of between 0.50 and 0.63 as directed by the manufacturer. The prepared suspensions for each of the isolates were individually placed in the identity (ID) and antimicrobial susceptibility test cassette (AST) for assay using Gram-negative (GN-ID AST) cards.

### 2.3. Antimicrobial Susceptibility and Minimum Inhibitory Test

The tested antimicrobials were ampicillin (Amp), Augmentin (Aug), ampicillin-sulbactam (SAM), piperacillin/tazobactam (Ptz), ceftazidime (CAZ), Ceftriaxone (Cxm), cefoxitin (Cfx), Ceftazidime (Caz), cefuroxime (Cxm), Amikacin (Amk), cefepime (Pime), aztreonam (Azt), imipenem (Imp), meropenem (Mer), gentamicin (Gm), tobramycin (Tob), ciprofloxacin (Cip), levofloxacin (Levo), tigecycline (Tig), colistin (Cs), tetracycline (Tet) trimethoprim/sulfamethoxazole (TMP/SMX), Tazocin (TZP), minocycline (Min), and ticarcillin/clavulanic acid (TIC/CLV).

Minimum inhibitory concentration (MIC) was ascertained with the Vitek 2 Automated System, and results were interpreted according to CLSI [12] guidelines. Standardized international terminologies of the Centers for Disease Control and Prevention (CDC) and the European Centers for Disease Prevention and Control were used to define multidrug resistance (MDR), extensive drug resistance (XDR), and extended-spectrum-beta-lactamase (ESBL) positive bacteria strains. Also, carbapenem-resistant isolates were categorized as carbapenem-resistant enterobacteriaceae (CRE).

### 2.4. Detection and Confirmation of ESBLs

Extended-spectrum beta-lactamases (ESBLs) isolates were detected by the Vitek 2 Automated System. Phenotypic assay was as recommended by CLSI [13] using the combined disc test (CDT). ESBL bacteria were seeded in Muller–Hinton agar, and discs of 30 µg ceftazidime (CAZ) and 30 µg cefotaxime (CTX) combined with 10 µg clavulanic acid (CLA) were incubated aerobically for 24 h at 37 °C. The results were interpreted based on a single individual test against ceftazidime and cefotaxime, as well as separately in combination with clavulanate, based on CLSI [13] guidelines.

### 2.5. Statistical Analysis

Data were collected and stored in Excel sheets. Data on the distribution of age are present as means and medians calculated with Excel data analysis. The data on age distribution, antimicrobial resistance, or sensitivity are presented as percentages, while graphs were computed using an Excel sheet and with GraphPad Prism 110.0(84), (San Diego, CA, USA). MedCalc Software Ltd. Comparison of two rates (https://www.medcalc.org/en/calc/rate_comparison.php Version 23.4.9; accessed 7 March 2026) was used to compare significance between percentages, with statistical significance taken as *p* < 0.05.

## 3. Results

### 3.1. Demographics, Sample Types, and Bacteria Pathogens

**Figure 1:**
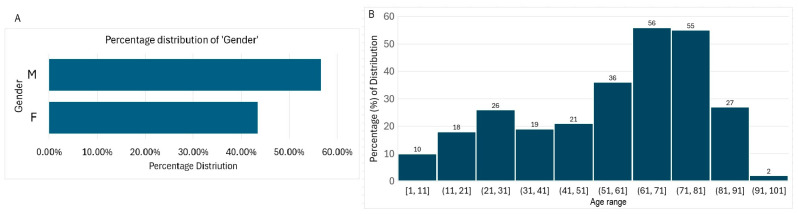
Demography, showing the gender (**A**) and age group distribution of patients (**B**). M = males; F = females.

A total of 270 Gram-negative bacteria isolates that formed part of patient care for males (58.27%) and females (41.73%) were used for the investigation (Figure 1A). The ages ranged from less than 1 year to 98 years old, with a mean age of 56.34 and a median age of 63 (Figure 1B). The most represented age group was the 71–81-year-olds, and the least represented age group was the 91–100-year-olds.

**Figure 2:**
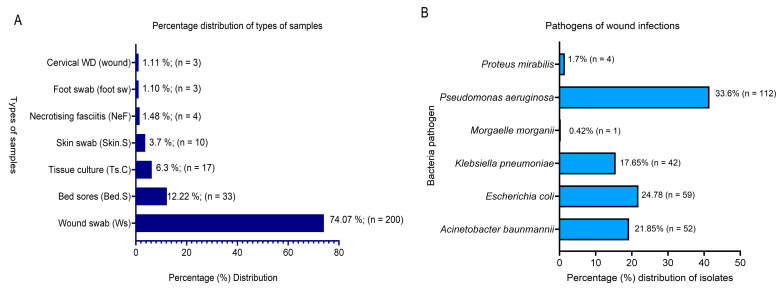
Sources of isolated wound-infection bacterial pathogens showing: (**A**) percentage sample distribution, (**B**) bacteria pathogens isolated from infected wounds.

For sample types, the majority were wound swabs (74.07%; n = 200), followed by bedsore samples (12.22%; n = 33). Other isolates were from tissue cultures (6.3% n = 17), skin swabs (3.7%), necrotizing fasciitis (1.48%; n = 4), foot swabs (1.10%), and cervical wounds (1.11%) (Figure 2A).

The bacterial pathogens associated with these infections were *Pseudomonas aeruginosa* (33.6%; n = 112), *Escherichia coli* (24.78% n = 59), *Acinetobacter baumannii* (21.85%; n = 52), *Klebsiella pneumoniae* (17.65%; n = 42), *Proteus mirabilis* (1.7%; n = 4), and *Morganelli morganii* (0.41%; n = 1) and the results are displayed in Figure 2B. Of the total number of bacteria pathogens (n = 270; 100%), 87 (32.22%) were confirmed using ESBL producers, constituting 51 *E. coli* isolates, 35 *K. pneumoniae,* and 1 isolate of *P. mirabilis*. Additionally, four of the isolates were classified as carbapenem-resistant enterobacteriaceae (CRE) isolates.

Samples originated from hospital wards (74.44%), intensive care units (ICU, 14.81%), emergency rooms (ER, 8.15%), and outpatient departments (OPD, 2.6%). This showed that the bacterial isolates, which formed part of the diagnosis for patient care, were from patients with comorbidities (Table 1).

### 3.2. Consolidated Antimicrobial Susceptibility Profile of the Isolates and ESBLs

**Figure 3:**
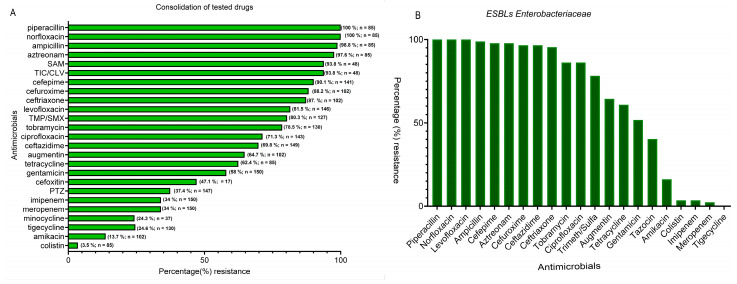

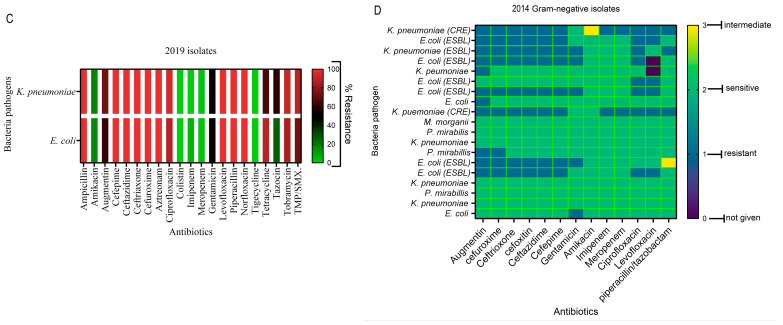
(**A**,**B**): consolidation of antimicrobial resistance to antibiotics for all bacterial isolates (**A**) and identified ESBL isolates (**B**). (**C**,**D**): Displays different levels of percentage resistance (**C**) to antibiotics for the period of 2019 and Gram-negative bacterial isolates susceptibility for the 2014 period (**D**). SAM = ampicillin-sulbactam; PTZ = piperacillin/tazobactam; TIC/CLV = ticarcillin-clavulanic acid; TMP/SMX = trimethoprim-sulfamethoxazole.

The results presented in Figure 3A are a consolidation of the percentage resistance of the tested antibiotics by the isolates. Overall sensitivity by the isolates was highest with colistin, while the next strongest performer was amikacin (86.3%). Carbapenems (meropenem, imipenem) exhibited an overall susceptibility of 66%, while fluoroquinolones and most of the cephalosporins performed poorly (Figure 3A). Also analyzed was a consolidation of antimicrobial susceptibility patterns for ESBL isolates. Results showed high resistance (91.95%) to third-generation cephalosporins (ceftazidime and/or ceftriaxone and aztreonam) while being susceptible to the carbapenems (imipenem and meropenem). Generally, for this group of isolates, the complete antimicrobial susceptibility profile showed the highest percentage resistance for piperacillin, norfloxacin, and levofloxacin (100%). Also high was resistance to cefepime (97.7%) and the cephalosporins in general. For the ESBL producers, the most active antibiotics with the lowest resistant percentages were tigecycline (0.0%), meropenem (2.3%), imipenem (3.45%), colistin (3.45%), and amikacin: 16.1% (14/87), as shown in Figure 3B. However, 100.0% of isolates met the MDR definition with resistance to three or more antimicrobial classes.

### 3.3. Antimicrobial Assay of Bacteria Species and the Year of Isolation

The tested antimicrobials differed in terms of the years of isolation; thus, resistance analyses were treated separately, comparing them according to bacteria species, year of isolation, and analysis based on antibiotic groupings.

#### 3.3.1. *Klebsiella pneumoniae*, *Escherichia coli*

In this group, isolates collected in 2014 and 2019 were used for the analysis. Results showed ESBLs *E. coli* and *K. pneumoniae* (2019 collection) were highly resistant to fluoroquinolones (ciprofloxacin, levofloxacin/norfloxacin) and penicillins (ampicillin/piperacillin) compared with 2014 isolates. Carbapenems (imipenem/meropenem), colistin, and tigecycline remained the most active across species, with amikacin maintaining moderate activity as compared to gentamicin (Figure 3C).

A slight difference is seen among the isolates collected 5 years earlier (2014), with a display of varying resistance profiles of carbapenem resistance (CRE), ESBL, and non-ESBLs, as displayed in Figure 3D. Generally, β-lactam/β-lactamase inhibitor (Augmentin) demonstrated the highest resistance (57.9%), while the fluoroquinolones and several cephalosporins displayed about 42% resistance. Thus, the best overall activity in terms of isolate susceptibility was by aminoglycoside (amikacin) with 94.7% and carbapenems (89.5%). The CRE-tagged *K. pneumoniae* stands out with resistance to both carbapenems. The majority of ESBL isolates cluster and show resistance to third and fourth generations of cephalosporins and to fluoroquinolones. Amikacin isolates again remain largely susceptible (94.7%). However, CRE exhibited higher resistance in number to the tested antibiotics as compared to the ESBLs and non-ESBLs isolates. The non-ESBL *K. pneumoniae* strains in this group were highly susceptible to tested drugs, showing green across in the heatmap (Figure 3D). For the other isolates, *M. morganii* and *P. mirabilis* exhibited a high susceptibility (15.4–100%).

#### 3.3.2. *Acinetobacter baumannii*

**Figure 4:**
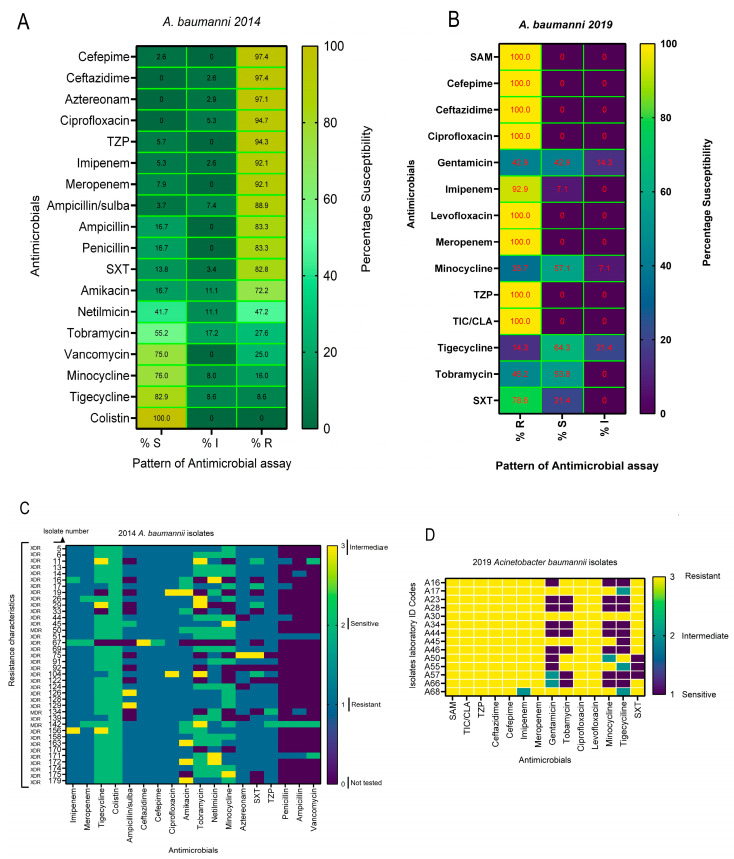
(**A**,**B**): Heatmaps of resistance pattern for 2014 and 2019 along with susceptibility of *A. baumannii* isolates. (**A**) 2014 and (**B**) 2019. TZP = piperacillin/tazobactam; TIC/CLV = ticarcillin-clavulanic acid; SAM = ampicillin-sulbactam; trimethoprim-sulfamethoxazole SXT. (**C**,**D**): Demonstrates resistance pattern for the individual isolates of 2014 (**C**) and 2019 (**D**). XDR = Extensive drug-resistance.

For *A. baumannii* wound-pathogen isolates, those collected in the years 2014 and 2019 were also included in this investigation. The results displayed in Figure 4A,B are the heatmap comparing the resistance profile of these isolates. For the 2014 *A. baumannii* isolates, there was a high β-lactam resistance with rates of 97.4% to third/fourth-generation cephalosporins (ceftazidime, cefepime) and aztreonam (97.1%), showing a near-complete state of non-susceptibility. In the case of fluoroquinolones and β-lactam/β-lactamase inhibitors, including ciprofloxacin, there is 100% non-susceptibility (R/I), and a high resistance to piperacillin/tazobactam (94.3%) was also observed. The carbapenems (imipenem and meropenem) showed a resistance of 92% each. A mixed resistance profile is displayed among aminoglycosides (amikacin resistance ~72%, tobramycin ~28%, and netilmicin 47%), demonstrating variability across agents in this class of antibiotics. The highest susceptibility is seen in the glycyclcycline–tetracycline class of antimicrobial agents (tigecycline and minocycline (≥76–83% S)), suggesting relative susceptibility activity in this set (Figure 4A).

Also, for the isolates collected in 2019, a very high resistance to β-lactams, including carbapenems (100% each for ampicillin/Sulbactam, cefepime, ceftazidime, meropenem), imipenem (92.9%), and fluoroquinolones (ciprofloxacin 100%) is seen among *A. baumannii* isolates. Resistance by drug family for the 2019 isolates were 100% for Penicillins/β-lactamase inhibitors, cephalosporins, carbapenems (driven by meropenem; imipenem shows 92.9% R and 7.1% I), and fluoroquinolones. The high resistance rates across β-lactam and fluoroquinolone families suggest extensive drug-resistant patterns. However, aminoglycosides and glycylcycline-tetracyclines (notably tigecycline and minocycline) retain some susceptibility (42.9% each). In comparison, a 78.6% resistance to trimethoprim-sulfamethoxazole (TMP-SMX) is also seen as high. All the isolates were sensitive to colistin (100%) with 0% resistance (Figure 4A,B).

The resistance burden per isolate is demonstrated in Figure 4C,D. Across all antibiotics tested per isolate, the mean proportion resistant is 0.79 (79%), with a range of 0.64 to 1.00, and an interquartile interval (25–75%) of 0.71–0.86. This indicates that most isolates are resistant to a large majority of agents tested. The burden of MDR/XDR is seen to be high across the isolates, with one of them (A30) resistant to all the tested antimicrobials (Figure 4D).

#### 3.3.3. *Pseudomonas aeruginosa*

**Figure 5:**
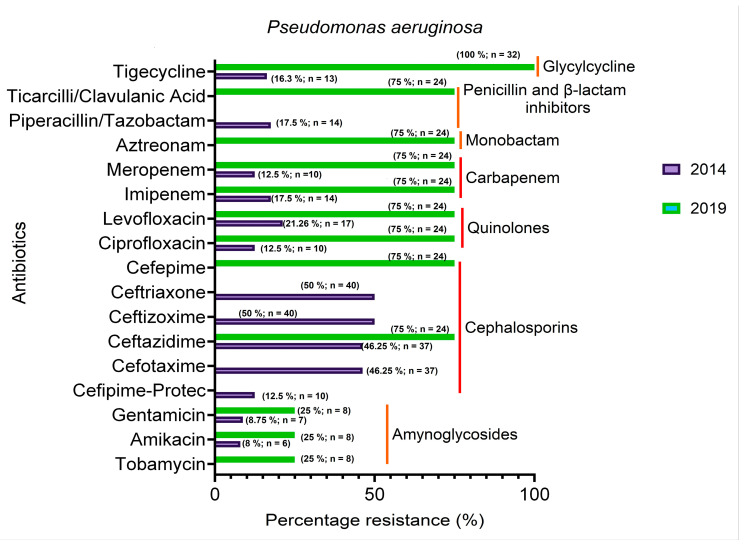
Showing different *Pseudomonas aeruginosa* isolates susceptibility to tested antibiotics for the 2014 and 2019 periods. Red color lines represent the categories of antibiotics.

Figure 5 represents the levels of percentage resistance of *P. aeruginosa* to tested antibiotics, comparing between 2014 and 2019 isolates. Results show that in 2014, most of the isolates were susceptible to the tested antibiotics, apart from cephalosporins. After five years (2019), the trend reversed, showing the majority of the isolates being resistant. However, aminoglycosides still retained their activity after 5 years (2019). In some instances, XDR was observed among the isolates. Overall results demonstrate that after a 5-year period, about 75% of the isolates of the bacteria species have become resistant to most of the antibiotics used for their management.

### 3.4. Minimum Inhibitory Concentration of Antibiotics and ESBL Gene Carriage

The results in Table 2 demonstrate the minimum inhibitory concentration of the tested antibiotics interpreted according to the guidelines of CLSI [12]. Results for resistance, intermediate, and susceptible values are displayed for the antibiotics, while there are none for amoxicillin (*K. pneumoniae* is intrinsically resistant to the antibiotics).

Table 2 shows the results of the ESBL combined disc test interpreted according to the guidelines of CLSI [13]. 

All the isolates tested for the confirmatory test had been identified by Vitek Compact 2 (BioMérieux, Marcy L’Etoile, France). Of the 51 *E. coli* isolates challenged for CTX/CLA or CAZ/CLA, 47 (92%) and 43 (84%) were confirmed significantly to be ELSB producers as compared to those in which they were not detected. Also, for *K. pneumoniae*, a significantly high number of the isolates challenged were confirmed as ESBL producers with CAZ/CLA (94%) and CXT/CLA (89%) and the results are presented in Table 3.

### 3.5. Antimicrobial Resistance Assay Based on Source of Bacteria Isolation

**Figure 6:**
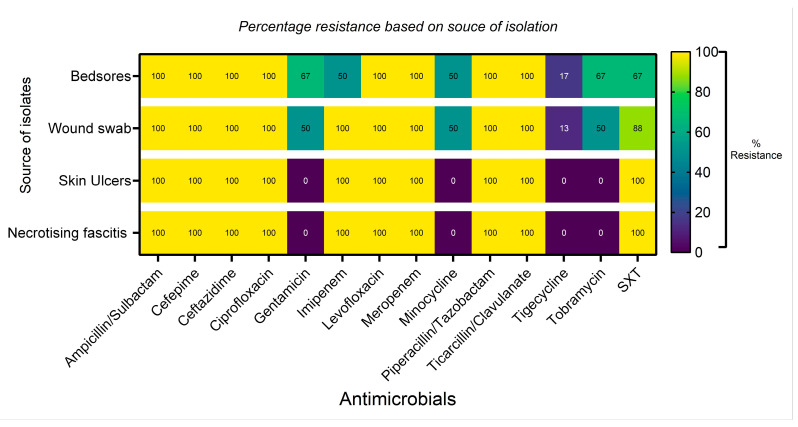
Antimicrobial resistance outlook based on the source of the bacteria pathogen. SXT = trimethoprim/sulfamethoxazole.

Resistance to antibiotics, as displayed by the source of isolation, shows extensive (100%) β-lactam and fluoroquinolone (penicillin/β-lactam inhibitors, cephalosporins, carbapenems, and fluoroquinolone) resistance across the regiments (Figure 6). Better antimicrobial activity is seen across all with tigecycline.

## 4. Discussion

The investigation shows that wound infections cut across gender and different age groups, while being highest among the middle-aged and the elderly (51–81), in findings that are similar to those of an earlier report [4]. The reasons attributed to this high incidence in the stipulated age group are the possibility of such patients presenting with other comorbid health conditions that lowers ability to ward off infections due to weakened immunity [14]. The fact that the least prevalent age group was patients in the range of 90 years of age and above could mean there is generally a smaller number of such patients. Besides this, there are other factors contributing to high antimicrobial resistance that are thought to be linked to the age of patients. Due to elderly patients’ comorbidities and weakened immune system, the elderly are thought to be unable to fight off infections in general. Subsequently, the high percentages of resistance as seen in this report could also be related to the high number of the elderly population from which bacteria isolates originated. Also, all isolated bacteria pathogens were from patients who had other comorbidities that could have necessitated the wide use of antibiotics prior to developing a wound infection. In addition to these, available literature supports the fact that antimicrobial resistance is considered a global public health concern, especially among the elderly and in males [1]. Worthy of note is that there were more males in this investigation than females, with such opinions supported by other researchers [1,15]. Also, the findings here are in consonance with those of previous reports [16,17].

Likewise, the Gram-negative bacteria pathogens included in this report are similar to those of other researchers, as they are of higher risk in causing recalcitrant wound infections [18]. However, the pattern by which bacteria species are most commonly encountered differs. Generally, though, Gram-negative bacteria are reported to be principally associated with wound infections in Saudi Arabia [19]. The highest number of isolates associated with wound infections was those of *P. aeruginosa,* followed by *E. coli*, *A. baumannii,* and *K. pneumoniae*. Some researchers [20,21] place the trend of predominant Gram-negative bacteria (GNB) to be *P. aeruginosa*, *K. pneumoniae,* and *E. coli*. However, *E. coli* was the most prevalent in a recent report from Indonesia [22], followed by *K. pneumoniae* and *P. aeruginosa*. While all findings established the involvement of the listed bacteria pathogens in wound infections, specific patterns regarding their frequency are not specific but do differ; the reasons could be attributed to regional and global differences.

Also, the reasonable percentage of *A. baumannii* associated with wound infections in this report is not unusual, as the bacterium is listed as one of the pathogens of wound infections [22], as well as being widely spread in healthcare settings [23] while being linked to outbreaks in trauma patients [24,25].

In terms of available suitable antibiotics for therapeutic options, results here further emphasized how limited the numbers are for the management of bacterial wound infections. The high percentage resistance that did not improve with subsequent years points to the public health problem of hard-to-treat bacteria pathogens in the region of the present study and globally too. Additionally, the fact that the ESBL isolates are susceptible to only five of the tested antibiotics (colistin, tigecycline, imipenem, meropenem, and amikacin), being the strongest performers, indicates that there are no exceptions to this trend. In agreement with the findings here are those of a recent report in Saudi Arabia, where researchers attributed high antibiotic resistance to the fact that management of wound infections is initiated empirically with antibiotics [1].

In terms of bacteria species, the time of isolation showed differences in the tested antibiotics for *K. pneumoniae* and *E. coli* for the years 2014 and 2019. While percentage resistance was seen to be higher among the 2019 isolates, amikacin and the carbapenems remained the most potent antibiotics regardless of year of isolation, in findings that are similar to those of an earlier report [1]. There is the possibility that these are the same strains of bacteria circulating in the region. Moreover, the high cephalosporin resistance by both *E. coli* and *K. pneumoniae* observed here is in harmony with other reports from Saudi Arabia and other regions [21,26]. However, the antibiotic-susceptible strains of *P. mirabilis* as seen here are different from those of an earlier report in Saudi Arabia [1]. Differences can be credited to factors that could be due to the number of tested isolates, which were quite few in this investigation, among other factors, such as the method of isolation and the tested antimicrobials.

One of the needs for continual surveillance is the resistance pattern displayed by *A. baumannii* isolates in this investigation. The high resistance to the carbapenems is consistent with this bacteria species, in view of the fact that while resistance to imipenem remained high and did not change over a gap of five years, the 2019 isolates were completely resistant to meropenem. This is in line with the 100% resistance observed previously [4], while slightly higher than those of other reports [9,26]. This is why this opportunistic bacteria pathogen is included among the ESKAPE list. Colistin and tigecycline might currently be the choice drugs, but probably not for long due to evolving resistance to these last-line drugs [27]. This is because while the isolates of 2014 were XDR, suitable therapeutic options could have been the aforementioned antibiotics. However, with PDR seen in 2019 isolates, there will be a need for other new therapeutic options. The disparities in suitable and available therapeutic options make comparisons of precise antibiotic susceptibility difficulty, not only regionally but across the world [4].

In the case of *P. aeruginosa,* while earlier (2014) collected isolates were susceptible strains (SS), the pattern was not maintained as later (2019) isolates were MDR. However, suitable therapeutic options remained the same for aminoglycosides (amikacin, tobramycin, and gentamicin) in findings that are similar to an earlier report [1]. Also, in line with the findings here are those of Momenah et al. [28] who reported high resistance to cephalosporins, quinolones, and carbapenems. While such similarities could be a reflection of the phenotypic strains circulating in the region, variations in patterns of susceptibility as reported by researchers could differ based on study conditions, the environmental impacts, tested antibiotics, and differing conditions of bacteria isolations [29].

## 5. Conclusions

This investigation into Gram-negative bacteria wound-infection pathogens showed that *P. aeruginosa* seems to be the most dominant compared to those of *E. coli*, *K. pneumoniae*, and *A. baumannii*. Also, suitable therapeutic options remain minimal due to high antibiotic resistance displayed by the isolates against tested antibiotics. While the suitability of amikacin, imipenem, and meropenem for isolates of *K. pneumoniae* and *E. coli*, irrespective of the year of isolation, the pattern was different for *A. baumannii* infections. For this bacteria species, the suitability of antibiotics changed with the year of isolation. While colistin and tigecycline were suitable as therapeutic options for 2014 *A. baumannii* wound isolates, this was not the same in the case for those of 2019. One similarity between the isolates, irrespective of the year of isolation, is the XDR characteristic, which justifies this opportunistic pathogen being classified as an ESKAPE in need of urgent new antibiotics. In the situation of *P. aeruginosa*, aminoglycosides (amikacin) remained appropriate as therapeutic options regardless of the year of sample collection. There is, however, a consistently high resistance to antibiotics by the bacteria pathogens associated with wound infections, as has been shown in this study, thus necessitating the need for continual surveillance for clinicians and healthcare providers in order to optimize patient care cost-effectively. The study also highlighted knowledge that could help in minimizing risk factors associated with wound infections.

## Figures and Tables

**Table 1:** Showing different bacteria isolates and their source of origin.

Bacteria Pathogen	Wards	ICU	OPD	ER	Grand Total
*P. aeruginosa*	90	12	0	10	112
*E. coli*	44	4	5	6	59
*K. pneumoniae*	25	13	2	2	42
*A. baumannii*	40	8	0	4	52
*M. morganii*	1	0	0	0	1
*P. mirabilis*	1	3	0	0	4
Total n; (%)	201; (74.44%)	40; (14.81%)	7; (2.6%)	22; (8.15%)	270; (100%)

n = number; ICU = intensive care unit; OPD = outpatient department; ER = emergency room.

**Table 2:** Minimum inhibitory concentrations of tested antibiotics.

Antimicrobials	Resistant	Sensitive	Intermediate
Amoxicillin	-	-	-
Ampicillin	16, ≥32	-	-
Amoxicillin/Clavulanic Acid	≥32	2, 4, 8	16
Ampicillin/Sulbactam	4, 16, ≥32	-	-
Piperacillin/Tazobactam	≥128	4	-
Cefalotin	≥64	2, 8	-
Cefoxitin	≥64	4	-
Ceftazidime	≥64	1	-
Ceftriaxone	≥64	1	-
Cefepime	≥64	1	-
Imipenem	≥16	≤0.25	2
Meropenem	≥16	≤0.25	-
Amikacin	≥64	≤2	8
Gentamicin	≥16	≤1	-
Ciprofloxacin	2, ≥4	≤0.25, 1	-
Tigecycline	≥8	≤0.5, 2	-
Nitrofurantoin	128, 256	≤16	64
Trimethroprim/sulfamethoxazole	≥320	≤20	-
Ticarcillin/clavulanic acid	≥128	16	-
Aztreonam	32, ≥64	4	-
Tobramycin	≥16	≤1	-
Levofloxacin	≥8	1	-

- = none; *Klebsiella pneumoniae* is intrinsically resistant to amoxicillin lactamases.

**Table 3:** Displays the results of the double-disc diffusion test for ESBL-producing isolates.

Bacteria Species (Number)		No. Positive (n/%)	No. Negative (n/%)	*p*-Value
*E. coli* (51)	CTX/CLA	47 (92)	6 (8.0)	0.000173 *
	CAZ/CLA	43 (84.3)	10 (15.7)	0.00019 *
*K. pneumoniae* (36)	CTX/CLA	32 (89)	5 (11)	0.00278 *
	CAZ/CLA	34 (94)	3 (6)	0.000595 *

* represents a significant difference between positivity and negative combined disc diffusion test for both *E. coli* and *K. pneumoniae* ESBL isolates. (Statistically significant calculator—TGM Research product used for the statistical analysis). https://tgmresearch.com/tools/statistical-significance-calculator.html. (accessed 27 February 2026).

## Data Availability

The data presented in this study are available on request from the corresponding author by appropriate request.

## Abbreviations

The following abbreviations are used in this manuscript:

MDR	Multidrug resistance
XDR	Extensive drug resistance
PDR	Pan drug resistance
AST	Antimicrobial susceptibility test
ESBLs	Extended-spectrum lactamases
ESKAPE	(*Enterococcus faecium*, *Staphylococcus aureus*, *Klebsiella pneumoniae*, *Acinetobacter baumannii*, *Pseudomonas aeruginosa*, and *Enterobacter* spp.)
(CRE)	Carbapenem-resistant enterobacteriaceae

## References

[1] Khalid F., Poulose C., Farah D.F.M., Mahmood A., Elsheikh A., Khojah O.T. (2024). Prevalence and Antimicrobial Susceptibility Patterns of Wound and Pus Bacterial Pathogens at a Tertiary Care Hospital in Central Riyadh, Saudi Arabia. Microbiol. Res..

[2] Zhou L., Zheng H., Liu Z., Wang S., Liu Z., Chen F., Zhang H., Kong J., Zhou F., Zhang Q. (2021). Conductive Antibacterial Hemostatic Multifunctional Scaffolds Based on Ti3C2Tx MXene Nanosheets for Promoting Multidrug-Resistant Bacteria-Infected Wound Healing. ACS Nano.

[3] Sen C.K. (2019). Human Wounds and Its Burden: An Updated Compendium of Estimates. Adv. Wound Care.

[4] Alharbi A.S. (2022). Bacteriological Profile of Wound Swab and Their Antibiogram Pattern in a Tertiary Care Hospital, Saudi Arabia. Saudi Med. J..

[5] Ibrahim M.E. (2019). Prevalence of Acinetobacter Baumannii in Saudi Arabia: Risk Factors, Antimicrobial Resistance Patterns and Mechanisms of Carbapenem Resistance. Ann. Clin. Microbiol. Antimicrob..

[6] Malaekah H.M., Alotaibi A.E., Alsebail R.A., Alelawi G.T., Alsarrani R.H., Banjar W.M. (2021). Wound Care Knowledge and Perception of the Saudi General Population in Riyadh Region. Adv. Wound Care.

[7] Robert A., Al Dawish M., Braham R., Musallam M., Al Hayek A., Al Kahtany N. (2016). Type 2 Diabetes Mellitus in Saudi Arabia: Major Challenges and Possible Solutions. Curr. Diabetes Rev..

[8] Li S., Renick P., Senkowsky J., Nair A., Tang L. (2021). Diagnostics for Wound Infections. Adv. Wound Care.

[9] Puca V., Marulli R.Z., Grande R., Vitale I., Niro A., Molinaro G., Prezioso S., Muraro R., Di Giovanni P. (2021). Microbial Species Isolated from Infected Wounds and Antimicrobial Resistance Analysis: Data Emerging from a Three-Years Retrospective Study. Antibiotics.

[10] Banawas S.S., Alobaidi A.S., Dawoud T.M., AlDehaimi A., Alsubaie F.M., Abdel-Hadi A., Manikandan P. (2023). Prevalence of Multidrug-Resistant Bacteria in Healthcare-Associated Bloodstream Infections at Hospitals in Riyadh, Saudi Arabia. Pathogens.

[11] Baraka M.A., Alboghdadly A., Alshawwa S., Elnour A.A., Alsultan H., Alsalman T., Alaithan H., Islam M.A., El-Fass K.A., Mohamed Y. (2021). Perspectives of Healthcare Professionals Regarding Factors Associated with Antimicrobial Resistance (AMR) and Their Consequences: A Cross-Sectional Study in Eastern Province of Saudi Arabia. Antibiotics.

[12] CLSI (2020). Performance Standards for Antimicrobial Susceptibility Testing.

[13] Clinical Laboratory Standards Institute (2012). Methods for Dilution Antimicrobial Susceptibility Testing for Bacteria That Grows Aerobically.

[14] Bajaj V., Gadi N., Spihlman A.P., Wu S.C., Choi C.H., Moulton V.R. (2021). Aging, Immunity, and COVID-19: How Age Influences the Host Immune Response to Coronavirus Infections?. Front. Physiol..

[15] Akhavizadegan H., Hosamirudsari H., Pirroti H., Akbarpour S. (2020). Antibiotic Resistance: A Comparison between Inpatient and Outpatient Uropathogens, Islamic Republic of Iran. East. Mediterr. Health J..

[16] OECD (2018). Stemming the Superbug Tide: Just A Few Dollars More.

[17] Swami S.K., Banerjee R. (2013). Comparison of Hospital-Wide and Age and Location—Stratified Antibiograms of *S. aureus*, *E. coli*, and *S. pneumoniae*: Age and Location-Stratified Antibiograms. Springer Plus.

[18] Moges F., Eshetie S., Abebe W., Mekonnen F., Dagnew M., Endale A., Amare A., Feleke T., Gizachew M., Tiruneh M. (2019). High Prevalence of Extended-Spectrum Beta-Lactamase-Producing Gram-Negative Pathogens from Patients Attending Felege Hiwot Comprehensive Specialized Hospital, Bahir Dar, Amhara Region. PLoS ONE.

[19] Bandy A., A Wani F., H Mohammed A., F Dar U., R Dar M., A Tantry B. (2022). Bacteriological Profile of Wound Infections and Antimicrobial Resistance in Selected Gram-Negative Bacteria. Afr. Health Sci..

[20] Shimekaw M., Tigabu A., Tessema B. (2020). Bacterial Profile, Antimicrobial Susceptibility Pattern, and Associated Risk Factors among Patients with Wound Infections at Debre Markos Referral Hospital, Northwest, Ethiopia. Int. J. Low. Extrem. Wounds.

[21] El-Saed A., Balkhy H.H., Alshamrani M.M., Aljohani S., Alsaedi A., Al Nasser W., El Gammal A., Almohrij S.A., Alyousef Z., Almunif S. (2020). High Contribution and Impact of Resistant Gram-Negative Pathogens Causing Surgical Site Infections at a Multi-Hospital Healthcare System in Saudi Arabia, 2007–2016. BMC Infect. Dis..

[22] Prastiyanto M.E., Darmawati S., Daryono B.S., Retnaningrum E. (2024). Examining the Prevalence and Antimicrobial Resistance Profiles of Multidrug-Resistant Bacterial Isolates in Wound Infections from Indonesian Patients. Narra J..

[23] Peacock S.J., Parkhill J., Brown N.M. (2018). Changing the Paradigm for Hospital Outbreak Detection by Leading with Genomic Surveillance of Nosocomial Pathogens. Microbiology.

[24] Eryilmaz-Eren E., Yalcin S., Ozan F., Saatci E., Suzuk-Yildiz S., Ture Z., Kilinc-Toker A., Celik I. (2023). An Outbreak Analysis of Wound Infection due to Acinetobacter Baumannii in Earthquake-Trauma Patients. Am. J. Infect. Control.

[25] El-Kholy A.A., Elanany M.G., Sherif M.M., Gad M.A. (2018). High Prevalence of VIM, KPC, and NDM Expression among Surgical Site Infection Pathogens in Patients Having Emergency Surgery. Surg. Infect..

[26] Li L., Dai J., Xu L., Chen Z., Li X., Liu M., Wen Y., Chen X. (2018). Antimicrobial Resistance and Pathogen Distribution in Hospitalized Burn Patients. Medicine.

[27] Madu Emeka P., Ineta Badger L., Estrella E., Belgira An G., Ezzat Khal H. (2022). Investigation of Colistin and Polymyxin B on Clinical Extreme Resistant Enterobacteriaceae Isolates for Surveillance Purposes. Int. J. Pharmacol..

[28] Momenah A.M., Bakri R.A., Jalal N.A., Ashgar S.S., Felemban R.F., Bantun F., Hariri S.H., Barhameen A.A., Faidah H., AL-Said H.M. (2023). Antimicrobial Resistance Pattern of Pseudomonas Aeruginosa: An 11-Year Experience in a Tertiary Care Hospital in Makkah, Saudi Arabia. Infect. Drug Resist..

[29] Khan M.A., Faiz A. (2016). Antimicrobial Resistance Patterns of Pseudomonas Aeruginosa in Tertiary Care Hospitals of Makkah and Jeddah. Ann. Saudi Med..

